# Aluminum in plant: Benefits, toxicity and tolerance mechanisms

**DOI:** 10.3389/fpls.2022.1085998

**Published:** 2023-01-13

**Authors:** Raphael Ofoe, Raymond H. Thomas, Samuel K. Asiedu, Gefu Wang-Pruski, Bourlaye Fofana, Lord Abbey

**Affiliations:** ^1^ Department of Plant, Food, and Environmental Sciences, Faculty of Agriculture, Dalhousie University, Bible Hill, NS, Canada; ^2^ School of Science and the Environment, Memorial University of Newfoundland, Grenfell Campus, Corner Brook, NL, Canada; ^3^ Charlottetown Research and Development Centre, Agriculture and Agri-Food Canada, Charlottetown, PE, Canada

**Keywords:** soil acidity, aluminum toxicity, growth promotion, exclusion, root inhibition, organic acid, Aluminum tolerant crops

## Abstract

Aluminum (Al) is the third most ubiquitous metal in the earth’s crust. A decrease in soil pH below 5 increases its solubility and availability. However, its impact on plants depends largely on concentration, exposure time, plant species, developmental age, and growing conditions. Although Al can be beneficial to plants by stimulating growth and mitigating biotic and abiotic stresses, it remains unknown how Al mediates these effects since its biological significance in cellular systems is still unidentified. Al is considered a major limiting factor restricting plant growth and productivity in acidic soils. It instigates a series of phytotoxic symptoms in several Al-sensitive crops with inhibition of root growth and restriction of water and nutrient uptake as the obvious symptoms. This review explores advances in Al benefits, toxicity and tolerance mechanisms employed by plants on acidic soils. These insights will provide directions and future prospects for potential crop improvement.

## 1 Introduction

Increasing crop productivity and quality are fundamental for achieving global food and nutritional security. The attainment of such goals is significantly constrained by several environmental factors including soil acidity and its associated aluminum (Al) phytotoxicity. Globally, acidic soils have been a concern and the major theme for scientific research. Acidic soils include oxisols or ultisol which have a pH value below 5 and are widely distributed in many regions of the world (Kochian et al., 2015). Acidic soils are more prevalent in tropical and subtropical regions and account for 60% of their soils, and 50% of the world’s agricultural lands, which hold up to 80% of global vegetable cultivation (Sade et al., 2016; Slessarev et al., 2016). While most soil acidity in tropical and subtropical regions occurs naturally, anthropogenic factors have recently become a major contributor to soil acidity in those regions and other parts of the world (Parth et al., 2011; Pavlů et al., 2021). Such factors include long-term and indiscriminate use of synthetic fertilizers, imbalance of soil nutrient cycle, organic matter build-up, excessive uptake and leaching of basic cations (Parth et al., 2011; Sade et al., 2016). The impact of soil acidity on crops is compounded by metal toxicity with Al^3+^ being the major limiting factor (Kochian et al., 2015). Moreover, the destructive impact of soil acidity is further aggravated by climate change and the endless heightened use of synthetic chemicals for crop production (Bojórquez-Quintal et al., 2017; Bungau et al., 2021).

Al is the third most ubiquitous metal in the earth’s crust after oxygen and silicon. However, Al is neither required in biological systems and to date, no scientific evidence has proven its use in any biological processes in living organisms, which remains a biochemical enigma. The chemistry of Al interactions in soils is remarkably complex and still not fully understood by researchers, possibly due to the wide array of organometallic and multinucleated complexes and co-occurring ions in soils (Buchanan et al., 2015; Chauhan et al., 2021). In most soils, Al exists as non-toxic aluminum silicates and oxides to which plant roots are exposed and exhibit no deleterious effects (Buchanan et al., 2015). However, a decrease in soil pH below 5 facilitates Al solubility into monomeric forms (Al (OH)^2+^, Al^3+^, Al (OH)_2_^+^ and Al (OH)_4_^-^). Among these, the trivalent form (Al^3+^) is the most deleterious to plant growth and productivity because it stimulates a range of Al-related toxicity in most plants (Kochian et al., 2015; Silva et al., 2020).

Over the past decades, several studies have demonstrated the effect of Al on growth and productivity in several plant species (Kochian et al., 2015; Sade et al., 2016; Bojórquez-Quintal et al., 2017; Singh et al., 2017). Generally, most of these studies reported the toxic effects of Al and the tolerance mechanisms of plants (Awasthi et al., 2019; Borges et al., 2020; Fang et al., 2020), while a few reported beneficial effects on plant growth (Muhammad et al., 2019; Sun et al., 2020b). In recent years, significant genetic diversities in Al tolerance and novel sustainable strategies have been identified in several crop species due to the agronomic importance of crop cultivation on acid soils (Kochian et al., 2015). Such studies are crucial in advancing our knowledge and identifying Al-induced tolerant genes and their associated mechanism for better crop improvement, thus contributing to global food security. This review will critically explore advances in Al benefits, toxicity and tolerance mechanisms employed by plants on acidic soils.

## 2 Al benefits and toxicity in plants

Generally, Al effects on plant growth and productivity have been viewed as a major threat to the attainment of global food security. Such effects have been demonstrated by earlier and more recent studies with root growth inhibition being the most obvious symptom of Al toxicity (Wang et al., 2016; Jaskowiak et al., 2018). On the other hand, stimulation of root and whole plant growth has been recognized as a beneficial effect of Al in several plant species (Arasimowicz-Jelonek et al., 2014; Muhammad et al., 2019; Liu et al., 2020). However, the levels of Al used in most of these studies are not clearly defined. While the levels of Al are expressed in its compound forms in some studies (Wang et al., 2016; Li et al., 2018b), others expressed it in a trivalent form which gives a better representation of the amount of Al plant roots are exposed to (Awasthi et al., 2017; Jaskowiak et al., 2018; Sun et al., 2020b). Moreover, the toxic or beneficial effect of Al on plant growth depends largely on the growing conditions, Al concentration and duration of exposure, plant species and physiological age (Huang et al., 1992; Bojórquez-Quintal et al., 2017; Aguilera et al., 2019; Ofoe et al., 2022). For example, low Al concentration of 0.25 and 0.5 mM did not affect *Trifolium* and tomato (*Solanum lycopersicum*) seedling root growth whereas high concentrations of 1.25 mM remarkably restricted root growth (Bortolin et al., 2020; Ofoe et al., 2022). In barley (*Hordeum vulgare*), low concentrations between 5-20 µM had no significant effect on root grow while concentrations of 40 and 60 µM reduced root growth. Similarly, exposure of plants to low Al doses for a short period inhibited root growth whereas no inhibition effect was noticed with higher Al concentrations for long-period exposure (Zhou et al., 2011). These suggest that different plant species have different response mechanisms to Al toxicity.

### 2.1 Benefits of Al to plant

Over the past decades, there has been overwhelming evidence published in several journals on the beneficial effects of Al on plants (Arasimowicz-Jelonek et al., 2014; Muhammad et al., 2019; Liu et al., 2020; Sun et al., 2020b). However, to date, no research has proven the biological significance of Al at the cellular level.

#### 2.1.1 Promotion of plant growth and metabolism

Al-induced plant growth promotion is often noticed in plants adapted to acidic soils, native species and Al hyperaccumulators (Arasimowicz-Jelonek et al., 2014; Bojórquez-Quintal et al., 2017; Sun et al., 2020b). Hyperaccumulators are indifferent to the concentration and duration of Al exposure and exhibit no toxic effect even at higher Al doses. For example, Rehmus et al. (2014) showed that a low Al dose of 300 µM enhanced the root biomass of *Tabebuia chrysantha* tree seedlings while a high concentration of 2400 µM induced an inhibitory effect. According to Pollard et al. (2014), two types of hyperaccumulators can be observed in plants: obligate and facultative. Obligate hyperaccumulators are plants that can only grow on metalliferous soils and are unable to survive once a particular metal is absent. In contrast, facultative hyperaccumulators can grow and survive regardless of the presence or absence of a given metal in soils. Applying these two types to Al hyperaccumulators might not be conclusive since Al growth studies have only been performed for fewer plant species (Schmitt et al., 2016).

Tree species such as *Camellia* spp (tea), *Symplocos paniculate, Quercus Serrata*, *Coffea arabica, Vochysia tucanorum* and *Melastoma malabathricum* are known to be Al-hyperaccumulators and can grow on acidic soils (Hajiboland et al., 2013a; Bojórquez-Quintal et al., 2014; Schmitt et al., 2016; Liu et al., 2020; Sun et al., 2020b; Bressan et al., 2021). In tea (*Camellia japonica*), Liu et al. (2020) demonstrated that treatment of 2-year-old plants with 0.5-1 mM Al enhanced root biomass *via* elongation and proliferation of lateral roots, chlorophyll content, net photosynthetic capacity and accumulation of nitrogen (N), phosphorus (P), iron (Fe), manganese (Mn), zinc (Zn), and copper (Cu) in the fine roots. Although the mechanism of Al-induced growth stimulation in *camellia* species remains elusive, it was suggested that the increased accumulation of nutrient elements could be an Al-induced mechanism (Liu et al., 2020). However, it is unclear how Al could facilitate the uptake of some macronutrients such as P and N which are known to be relatively unavailable under low soil pH. Similarly, Sun et al. (2020b) using five *C. sinensis* varieties showed that Al treatment enhanced new root growth in a dose-dependent manner while tea plants deprived of Al treatment exhibited damaged root tips and no new root formation. Root tip ultrastructural analysis revealed that Al is crucial for meristematic cell development and activities as root cells of Al-treated tea plants were dense with a large and noticeable nucleus compared to Al-deprived plants. Interestingly, Al localization examination indicated that Al is contained in the nuclei of root meristems and translocated to the cytosol upon removal which worsens DNA damage and suggests that Al could primarily function in tea plants root growth *via* DNA integrity maintenance (Sun et al., 2020b). Also, Bressan et al. (2021) showed that exposure of In *Vochysia tucanorum* seedling to 1110 µM Al exhibited increased root growth, root, stem and leaf biomass and conserved high photochemical performance and leaf gas exchange rates. Additionally, seedlings with no Al exposure showed no new root formation after 7 days and stopped growing with increased pre-existing roots necrosis and leaf chlorosis. Exposure of *M. malabathricum* seedlings to 0.5 mM Al considerably improved root and shoot growth, and relative dry weight *via* secretion of root mucilage which facilitates Al accumulation and improves nutrient (P, K, Ca and Mg) and water uptake (Watanabe et al., 2005; Watanabe et al., 2008b; Watanabe et al., 2008a). In *Q. serrata*, Al stimulates root elongation and activities and decreased root starch and sucrose content but increased glucose and ABA content in Al-treated roots (Moriyama et al., 2016). In the case of deciduous *S. paniculate*, it was reported that Al stimulates root growth and elongation in seedlings while saplings developed new twigs, leaves and roots thereby considerably enhancing the overall biomass (Schmitt et al., 2016).

**Figure 1:**
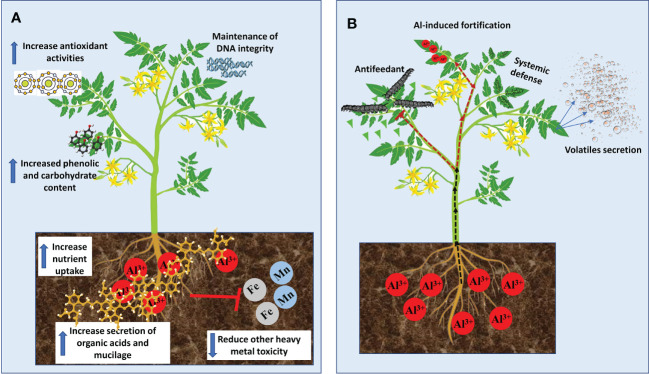
Benefits of Aluminum to plant growth and development. **(A)** Mechanisms of Al-mediated growth simulation. **(B)** Mechanism of Al-mediated protection against biotic stress.

Although most of the growth stimulation effects of Al in plants were reported in tree species that are adapted to acid soils, fewer studies have demonstrated such benefits in some economically important crops. For example, in maize (*Zea mays*) plants, low Al dose treatment inhibited root growth but significantly increased leaf growth rate in an Al exposure duration-dependent manner (Wang et al., 2015b). In rice (*Oryza sativa*) plants, Al enhanced root elongation (Famoso et al., 2011), chlorophyll and carotenoid contents as well as shoot height (Nhan and Hai, 2013). Similarly, Moreno-Alvarado et al. (2017) demonstrated using four rice cultivars exposed to 200 μM Al that Al considerably increased plant height, chlorophyll and sugar contents, root length and root fresh and dry biomass but did not affect amino acid and proline contents. In Al-tolerant soybean (*Glycine max*), 25 µM Al for 24, 36 or 48 h markedly increased callus and root growth (Du et al., 2010). Recently, it was reported in tomato (*Solanum lycopersicum*) that 500 μM Al stimulated total root length, hypocotyl and root surface areas as well as the overall total seedling fresh weight (Ofoe et al., 2022). Poschenrieder et al. (2015) suggested that two modes of Al-induce growth stimulation can be noticed in plant species; (1) immutable boost (long-term) in growth stimulated by Al in hyper-tolerant plants; and (2) momentary increase (short-term) in growth as observed in most laboratory studies. Despite the plant growth stimulation effects reported by several authors, it remains elusive the exact mechanisms underpinning such effects. Nevertheless, a few possible mechanisms have been proposed by some studies (**Figure 1A**). Evidence from Al-hyperaccumulators studies indicates that Al-mediated growth stimulations are closely related to increase nutrient uptake (Watanabe et al., 2005; Liu et al., 2020), activation of antioxidants and metabolic enzymes pathways (Hajiboland et al., 2013a; Xu et al., 2016), elevated secretion of mucilage and organic acids (Watanabe et al., 2008b; Watanabe et al., 2008a; Xu et al., 2016), maintenance of DNA integrity (Sun et al., 2020b) decreased other heavy metal toxicity (Hajiboland et al., 2013b) and increased accumulation of carbohydrate and phenolic compounds (Moriyama et al., 2016; Xu et al., 2016; Maejima et al., 2017).

#### 2.1.2 Mitigation of abiotic and biotic stresses

Studies on the beneficial effect of Al have focused on plant growth stimulation and enhancement of nutrient uptake while its positive effects on biotic and abiotic stresses have not been widely explored in the literature. Al has been reported to promote plant resistance to biotic (pathogens and herbivores) and abiotic stresses including nutrient deficiency and ion toxicity (Kaur et al., 2016; Bojórquez-Quintal et al., 2017). In tall fescue (*Festuca arundinacea*), Al enhanced plant growth and reduced the biomass and severity of white grubs (Coleoptera: Scarabaeidae) (Potter et al., 1996). Although the mechanism of such a defense response is unknown, it was suggested that Al accumulation in leaf tissues forms a sensory barrier that inhibits female insects from oviposition and thereby, reducing grubs population. In plant pathological studies, Al inhibits blast rot pathogen (*Thielaviopsis basicola Ferraris*) spores’ germination and vegetative growth (Meyer et al., 1994). Similarly, Al enhanced resistance to potato late blight pathogen (*Phytophthora infestans*) by restricting mycelia and sporangial germination (Andrivon, 1995) and inducing defence responses in susceptible potato plants (Arasimowicz-Jelonek et al., 2014). Such a protective mechanism was characterized by localized accretion of ROS (e.g. H_2_O_2_) in roots and systemic induction of nitric oxide and salicylic acid-dependent pathways in leaves. These correlated with pathogen-related gene expressions and protein activities (Arasimowicz-Jelonek et al., 2014). Although it remains unclear how Al mediates such responses, various ways have been presented (**Figure 1B**), which suggests that Al could be deployed as an effective strategy for devastating disease control.

Despite the increased solubility and availability of Al in acidic soils, the presence of other metals such as Mn and Fe are high and can induce a toxic effect in plants (Bojórquez-Quintal et al., 2017; Muhammad et al., 2019). Rice plants treated with 200 µM Mn alone affected shoot and root growth whereas the addition of 200 µM Al alleviated Mn toxicity (i.e., leaf chlorosis and necrosis) in rice seedlings (Wang et al., 2015d). Such Al-induced mitigation of Mn toxicity was enhanced by reduced Mn uptake in roots and subsequent accumulation in shoots due to changed membrane potential and modified cell membrane binding properties in rice roots (Wang et al., 2015d). Likewise, Muhammad et al. (2016) demonstrated that Al-induced antagonistic interaction with Mn and ameliorates Mn toxicity in three genotypes of barley (*Hordeum vulgare*). Moreover, toxic levels of Fe induce oxidative stress *via* excess production of ROS in plants which facilitate cellular damage and loss of functions (Zahra et al., 2021). Al-induced alleviation of Fe toxicity has been reported in tea (Hajiboland et al., 2013b), *M*. *malabathricum* (Watanabe et al., 2006) and rice plants (Alia et al., 2015). In these species, Al decreased root cell surface charges to restrict Fe uptake and translocation and hindered bronzing of leaves of treated plants. In hydroponic studies, Yang et al. (2016b) showed that exposure of tea plants to fluoride (F) inhibited the growth of shoots and roots while the addition of Al neutralized F toxicity by forming an Al-F complex to stimulate the growth of tea plants. Also, P deficiency is a major concern in acidic soils and Al has been reported to promote P uptake in plants (Li et al., 2016a). In maize, Al application induces the expression of genes that prevents inorganic P starvation (Maron et al., 2008), while Al up-regulated low P-responsive protein accumulation in citrus (*Citrus sinensis*) leaves. Such proteins include purple acid phosphatases, pyrophosphatases, phosphoenolpyruvate carboxylase, glycerophosphodiester phosphodiesterase and ribonucleases, which are known to play critical roles in plant adaptation to Pi-limitation by hydrolyzing organic P and/or (pyrophosphate) PPi to Pi, enhancing Pi acquisition and utilization, inducing Pi release from macromolecules and remobilizing Pi availability (Li et al., 2016a).

### 2.2 Al phytotoxicity

Al toxicity to plants is one of the major threats to crop productivity under acidic soils (Kochian et al., 2015). Al triggers a series of Al-induced phytotoxic syndrome which includes disruption of root growth and development, reduction in photosynthesis and plant growth, accumulation of reactive oxygen species (ROS) and damage of cellular and biochemical components.

#### 2.2.1 Inhibition of root growth and development

The primary symptoms of Al toxicity in plants are rapid inhibition of root growth and disruption of root morphology (Buchanan et al., 2015; Zhang et al., 2019a). Such reduction in root growth has been widely used as a marker in evaluating Al toxicity or Al tolerance plants (Awasthi et al., 2017). Root tips are the most sensitive part of the root system and respond to micromolar concentrations of Al (Huang et al., 2014b). It has been established that the root tip is the most prominent plant organ and the distal transition zone between the apical meristem and elongation zone of roots plays a critical role in sensing Al toxicity (Yang and Horst, 2015; Zhu et al., 2017; Zhu et al., 2019; Zhang et al., 2019b; Wu et al., 2022). This suggests that Al toxicity or tolerance mechanism should predominantly focus on root studies. Al-induced inhibition of root growth and disruption of root structure has been reported in several plant species and the timing of symptoms development varies from plant to plant (Yanık and Vardar, 2015; Balzergue et al., 2017; Amaral et al., 2018; Furlan et al., 2018; Agarwal et al., 2019; Chen et al., 2020; Ofoe et al., 2022). For instance, while inhibition of Al-sensitive maize root elongation was observed after 30 mins of Al exposure (Llugany et al., 1995), root growth inhibition of soybean occurred after 5 min of Al exposure (Kopittke et al., 2015). Al affects root tip cell division and elongation which results in abnormal cell organization and thickening of the root cell wall (Yan et al., 2018; Zhu et al., 2019). Al-induced root growth inhibition results from several interactions between Al^3+^ and cellular components of plant roots (Buchanan et al., 2015; Silva et al., 2020). Root cell walls are composed of pectin, cellulose and hemicellulose, which serve as a protective barrier against harmful environmental cues and are crucial for plant defence (Geng et al., 2017; Wu et al., 2022). These structural materials are rich in negatively charged phosphate and carboxylic groups, which can interact with Al ions (Geng et al., 2017). Increasing evidence revealed that Al initially targets the root epidermis and cortex and instantly binds to root cell walls which jeopardises cell wall integrity and functions including cell expansion and whole root growth (Yang and Horst, 2015; Geng et al., 2017; Zhu et al., 2017; Wu et al., 2022). However, the exact mechanism of Al-induced root growth inhibition remains elusive.

#### 2.2.2 Reduction of water and nutrient uptake

Exposure of plants to Al toxicity instigates water stress, particularly physiological drought that restricts plant capacity to acquire water and nutrients (Kochian et al., 2015; Buchanan et al., 2015). Such impedance in nutrient uptake is facilitated by Al-induced disruption of root cells, inhibition of root growth and reduction in root volume. Previous studies have provided compelling evidence that Al affects plasma membrane functions and thereby, regulating the flow of ions to important parts of the plant for physiological processes (Guo et al., 2007; Gupta et al., 2013). Moreover, the active transport of nutrients is triggered by hydrogen ion gradients which are mediated by proton antiporters located at the plasma membrane (Zhang et al., 2019a). Al binds to the negatively charged phospholipid bilayers of the plasma membrane which destabilizes membrane potential and inhibits H^+^-adenosine triphosphatase (H^+^-ATPase) proton exclusion activities. Consequently, this affects transport of nutrient ions including K^+^, NH_4_^+^, Mg^2+^ and Ca^2+^ (Gupta et al., 2013; Zhang et al., 2017a). In maize, Mariano et al. (2015) used a divided-root-chamber technique to reveal that Al altered nutrient uptake in the roots and considerably reduced the net uptake of Ca and Mg but not K content. Moustaka et al. (2016) reported that exposure of Al-sensitive wheat (*Triticum aestivum*) plant to increasing Al concentration (0–148 μM) resulted in decreased Ca and Mg content in leaf tissue. In pea (*Pisum sativum*), Kichigina et al. (2017) indicated that Al reduced K, Mg, Zn, Mn and S content in the root and shoots of Al-treated plants. Guo et al. (2018) showed that Al treatment did not only reduce N, P, K, Ca, Mg and S uptake but also decreased the relative water content of citrus root and leaves. In sugarcane, Al significantly reduced nutrient use efficiency of macro and micronutrients (Borges et al., 2020). Additionally, P deficiency is a major concern in acidic soils as Al has a high affinity to P and forms insoluble Al-P compounds in soils (Magalhaes et al., 2018). Numerous studies have reported that Al reduces P uptake and utilization in several plant species such as *Eucalyptus* (Teng et al., 2018), oat (*Avena sativa*) (Djuric et al., 2011), *Citrus grandis* (Guo et al., 2017) and soybean (Chen et al., 2019b). These studies indicate that Al toxicity results in plant nutritional imbalance, which can affect the growth and productivity of crops.

#### 2.2.3 Reduction of photosynthetic capacity

The impact of Al toxicity on plant photosynthesis has been studied extensively in several plant species (Yang et al., 2015; Yusuf et al., 2016; Guo et al., 2018; Cheng et al., 2020). In Al-sensitive barley, Al treatment markedly reduced chlorophyll content and fluorescence as well as gas exchange parameters including net photosynthetic rate, intercellular CO_2_ concentration, stomatal conductance and transpiration rate (Ali et al., 2011). In *Citrus*, Al-induced a decrease in chlorophyll pigment and altered chlorophyll a (Chla) fluorescence transient and fluorescence parameter which resulted in the overall reduction of leaf photosynthesis (Jiang et al., 2008; Guo et al., 2018). Similar effects of Al-induced reduction in total chlorophyll content, chlorophyll fluorescence and leaf photosynthesis rate were reported in maize (Zhao et al., 2017), rye (*Secale cereale*) (Silva et al., 2012), *Eucalyptus* (Yang et al., 2015), peanut (*Arachis hypogaea*) (Shen et al., 2014), cocoa (*Theobroma cacao*) (Ribeiro et al., 2013), alfalfa (*Medicago sativa*) (Cheng et al., 2020), rice (Phukunkamkaew et al., 2021) and highbush blueberry (*Vaccinium corymbosum*) (Cárcamo et al., 2019). Evidence revealed that the decrease in leaf photosynthesis under Al stress is indicative of the performance of photosystem II (PSII) (Jiang et al., 2008; Guo et al., 2018). Chlorophyll a fluorescence induction analysis of Al treated citrus leaves showed a significant reduction of the maximum quantum yield of primary photochemistry (Fv/Fm), maximum chlorophyll fluorescence (Fm), total PSII performance index, the quantum yield of electron transport and oxygen-evolving complex (Jiang et al., 2008). Moreover, such Al-induced impairment in the overall photosynthetic electron transport network from PSII was proposed as the major cause of reduction in leaf CO_2_ assimilation (Jiang et al., 2008). It was also suggested that Al toxicity could block electron transport and diminish PSII photochemistry thereby impeding leaf photosynthesis (Li et al., 2012).

#### 2.2.4 Oxidative stress and cellular damage

Rapid production and accumulation of ROS including superoxide (O_2_ **^·^** ^−^), hydrogen peroxide (H_2_O_2_), singlet oxygen (^1^O_2_) and hydroxyl (OH**^·^** ^−^) radicals are one of the most important alterations in cell metabolism of plants under Al stress (Huang et al., 2014b; Furlan et al., 2018). Imbalance ROS production occurs a few minutes after Al exposure, which induces oxidative stress in plants and facilitates the damage of cellular components such as nucleic acids, membrane lipids and proteins (Huang et al., 2014b; Guo et al., 2018; Yamamoto, 2019). Such ROS are generated in either the mitochondria, peroxisome or chloroplast in response to Al treatment (Shavrukov and Hirai, 2016; Turkan, 2018). Al-induced ROS accumulation has been reported in several plant species such as tomato (Borgo et al., 2020; Ofoe et al., 2022), rice (Awasthi et al., 2017; Awasthi et al., 2019; Bera et al., 2019), black gram (*Vigna mungo*) (Chowra et al., 2017), tea (Devi et al., 2020), soybean (Chen et al., 2019b), highbush blueberry (Cárcamo et al., 2019), *Trifolium* (Bortolin et al., 2020) and peanut (Huang et al., 2014a; Huang et al., 2014b). Despite its deleterious effect, ROS can perform a dual function in plants; acting as important signal molecules to regulate metabolic and physiological processes and activating the expression of antioxidant machinery for Al-stress mitigation (Turkan, 2018).

Moreover, it was previously established that Al induces excessive ROS production *via* two main mechanisms, (1) rapid activation of NADPH oxidase in the plasma membrane (Kawano et al., 2003) and (2) distortion of mitochondrial electron transfer pathways (Yamamoto et al., 2002). NADPH oxidase-mediated ROS production occurs immediately and halts within the first 20 s after Al exposure. However, mitochondrial dysfunction-mediated ROS production occurs several hours after Al exposure suggesting that Al could enter the cell and perturb the normal function of these organelles. These indicate that as Al binds to the plasma membrane it activates the initial ROS signal *via* NADPH oxidase-mediated pathways, which could possibly activate antioxidant pathways for initial ROS detoxification. Besides, Al enters the cell and distorts membrane organelles including the mitochondrion which results in cellular damage. Interestingly, several studies have shown that Al-induced-ROS generation facilitates lipid peroxidation, the most remarkable symptom of oxidative stress, which accelerates membrane loss and protein degradation, and ultimately results in programmed cell death (Singh et al., 2017; Yamamoto, 2019; Ofoe et al., 2022). Lipid peroxidation follows a chain of free radical reactions which results in the production of malondialdehyde (MDA), a highly reactive end product. In tomato plants, Al treatment enhances the production and accumulation of MDA and destabilizes membrane functions (Borgo et al., 2020; Ofoe et al., 2022). In tea plants, Devi et al. (2020) demonstrated that increasing Al doses increases ROS production which elicits lipid peroxidation and oxidation of macromolecules in roots and leaves of treated plants while in black gram, protein carbonylation was also observed (Chowra et al., 2017). Similarly, Al-induced ROS-mediated MDA accumulation was reported in rice (Ma et al., 2012), soybean (Chen et al., 2019b), tobacco (Yamamoto et al., 2002) and pea (Huang et al., 2014b).

#### 2.2.5 Nucleus and DNA damage

The maintenance of nuclear materials is crucial for plant survival under environmental stress. It has been reported that upon Al attachment with the cell wall and further penetration into the cell *via* the disruption of the plasma membrane, it interacts with nuclear structures which subsequently affect the integrity of DNA and chromosomes (Eekhout et al., 2017; Jaskowiak et al., 2018; Szurman-Zubrzycka et al., 2019). It is suggested that the DNA is the major cellular target of Al where it binds to the negatively charged phosphodiester backbone and leads to changes in DNA conformation from B-form to Z-form. This reduces DNA replication by increasing DNA firmness which results in difficulty in unwinding DNA (Gupta et al., 2013; Eekhout et al., 2017; Wei et al., 2021). In pea plants, Huang et al. (2014a) used DAPI-staining of root tip cells to reveal that increasing Al exposure time from 4-12 h disorganized root cells, compromised membrane integrity and the affected nuclei appeared squished, lobed and abnormal in shape. These resulted in DNA fragmentation and shortening and degradation of nuclear chromatin after 4 h exposure to 100 µM AlCl_3_. Similarly, a cytological study of *Pinus massoniana* roots indicated that Al toxicity disrupts cell division, which was characterized by physiological alteration of nucleoproteins and induction of four types of chromosomal aberrations including chromosomal adhesion, chromosomal fragmentation, c-mitosis, and chromosomal bridges (Zhang et al., 2014). Such Al-induced chromosomal aberration might be irreversible and could result in programmed cell death.

Moreover, in a study of Al-induced cell death in six cereal roots, Vardar et al. (2016) demonstrated that DNA fragmentation which is indicative of cell death was induced at 30 mins after 100 μM AlCl_3_ treatment. The Al-induced cell death was noticed in barley, triticale (*×Triticosecale*), oat and rye roots, but not in wheat and maize. These data suggest that wheat and maize might be comparatively more tolerant than the other plant species. Likewise, Jaskowiak et al. (2018) showed that Al significantly reduced cell mitotic activity, and stimulated micronuclei formation and disintegrated nuclei in barely root cells in a time-dependent manner. Also, the TUNEL test and flow cytometry analysis indicates that Al toxicity damages DNA, alters cell cycle and delays cell division in barley meristematic root cells. Although it is obvious that Al instigates disruption of cell cycles, it remains elusive whether such disruptions occur throughout the cell cycle and if it is species and region dependent as most studies focus on root tips. Additionally, monitoring cytotoxic changes in time and whether these changes could be reversed after Al withdrawal will help elucidate the Al-induced cell cycle effects.

Although the mechanism of how Al damages DNA remains unknown, loss-and-gain of function mutational analyses have provided insight into understanding the effect of Al on DNA double-strand breaks (DSBs) (Nezames et al., 2012; Sjogren et al., 2015; Sjogren and Larsen, 2017; Eekhout et al., 2017). Mutants in key DNA damage response (DDR) genes such as suppressor of gamma response 1 (SOG1), ataxia telangiectasia and RAD3 related (ATR), sensitive to UV 2 (SUV2) and aluminum Tolerant 2 (ALT2) have been shown to partially reverse growth inhibition in mutants defective in ABC transporter required for normal growth (Al-sensitive 3 (ALS3) under Al toxicity (Nezames et al., 2012; Sjogren et al., 2015; Sjogren and Larsen, 2017; Eekhout et al., 2017). ATR is an important DNA checkpoint regulator enlisted by SUV2 to strengthen single-stranded DNA because of a delay in replication fork movement, whereas ATM associates with ATR and functions in detecting DNA double-strand breaks (Hu et al., 2016; Szurman-Zubrzycka et al., 2019). SOG1 is a central DDR transcription factor that is phosphorylated by both ATR and ATM to modulate genes involved in DNA damage response (Yoshiyama et al., 2013). ALT2 is a WD-40 protein that is crucial for assessing DNA integrity (Nezames et al., 2012). Further studies in *Arabidopsis* and barley revealed that *atr* mutants exhibited improved root growth even at high Al concentrations (Szurman-Zubrzycka et al., 2019; Chen et al., 2019a). On the other hand, Sjogren et al. (2015) demonstrated that *atm* suppressed Al-hypersensitivity and increased DNA synthesis-dependent endoreplication levels in *als3* mutants. Similarly, Chen et al. (2019a) revealed that *atm* mutants compromised their recovery after exposure to high Al treatment. These suggest that Al does not interfere with DNA replication and that high Al dose may predominantly cause DSBs but not single strand breaks since ATM is important for sensing DSBs.

#### 2.2.6 Cytoskeleton disruption

Cytoskeleton primarily functions in various cellular processes including cell growth, cell differentiation, cell division and internal arrangement which contribute to root growth (Gardiner et al., 2012; Quezada et al., 2022). It consists of a network of microtubules, actin filaments and other related proteins which are potential targets for cytosolic Al toxicity (Gardiner et al., 2012; Sade et al., 2016). It has been well documented that Al destabilizes and/or delays microtubule cytoskeleton arrangements and alters tubules polymerization resulting in restriction of root growth (Amenós et al., 2009; Baranova et al., 2016). In *P. massoniana* root cells, Al exposure induces an aberrant formation of microtubule arrangement which was characterized by short microtubule fragments (Zhang et al., 2014). Moreover, increasing Al concentration for a longer duration severely perturbed the microtubule organization and performance of phragmoplast and mitotic spindle fibres which were attributed to extensive depolymerization of microtubules and actin filaments (Zhang et al., 2014). Fang et al. (2020) showed that the effect of Al toxicity on actin filaments organizations is Al concentration-dependent as 100 μM AlCl_3_ had no distinct effect on actin filaments, whereas treatment of *Malus domestica* pollen tubes with 600 μM AlCl_3_ triggers an abnormal adjustment of the actin filaments. Interestingly, transcriptomic analyses revealed that Al toxicity downregulated numerous genes involved in cytoskeleton metabolism in two citrus species roots (Guo et al., 2017) and leaves (Li et al., 2016a), and tea roots (Fan et al., 2019a). These studies suggested that cytoskeletal components in roots, leaves and pollen tube cells could be a primary target for Al toxicity and thereby, inhibiting cell growth and division. Nevertheless, it remains unclear how Al might interact with cytoskeletal elements. Furthermore, it was suggested that Al can perturb the total cytoskeleton dynamics by directly associating with cytoskeletal elements and/or indirectly altering cytosolic Ca^2+^ signalling networks that are critical in cytoskeletal stabilization (Amenós et al., 2009; Gupta et al., 2013; Sade et al., 2016).

## 3 Mechanisms of Al tolerance in plants

Plants are sessile and exposed to varying degrees of Al stress. However, most plants have evolved diverse mechanisms including physiological, biochemical and molecular mechanisms to cope and survive under Al toxicity. Such mechanisms are broadly grouped into two types: Al external exclusion mechanism which is aimed at avoiding Al from entering root cells; and symplastic or internal tolerance mechanism which allows entry of Al into root cells, detoxification and compartmentalization into subcellular compartments (Kochian et al., 2015; Kopittke et al., 2015; Sade et al., 2016; Bojórquez-Quintal et al., 2017; Furlan et al., 2020).

### 3.1 Al exclusion mechanism

#### 3.1.1 Exudation of organic compounds

**Figure 2:**
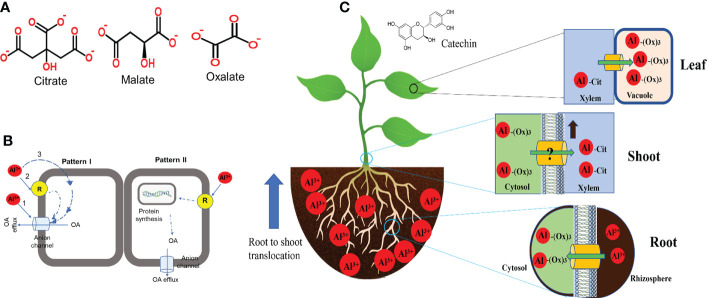
Al-induced modes of organic acid (OA) released and internal tolerance in plant roots. **(A)** Structure of the types of OA exudes by different plant species in response to Al exposure. **(B)** Proposed mode of Al-induced OA secretion (Modified from Ma et al. (2001)). **(C)** Internal detoxification and compartmentalization of Al in plants.

Organic acids (OA) efflux is the most characterised and well-documented mechanism in Al tolerance in plants. In response to the rhizotoxic effect of Al, most plant roots release deprotonated anions that bind Al at the rhizosphere to form non-toxic complexes and impede root entry (Kochian et al., 2015). The commonly identified OAs secreted by plants in response to Al exposure are citrate, malate and oxalic acids (**Figure 2A**) (Kichigina et al., 2017). Root exudation of OA is Al^3+^-dependent, and the type of OA secreted differs from plant to plant (**Table 1**), but large differences occur mainly in cereals (Schroeder et al., 2013; Bojórquez-Quintal et al., 2017). Citrate and malate are the most common OA efflux by several plant species, whereas oxalate secretion has only been identified in buckwheat (*Fagopyrum esculentum*) and Taro (*Colocasia esculenta*) (Buchanan et al., 2015). These OAs are components of the tricarboxylic acid cycle localised in the mitochondria, ubiquitous in all plant cells and exhibit different chelating abilities with Al ions (Brunner and Sperisen, 2013). Moreover, plant cells are rich in OAs but secretes specific ones in response to Al which suggest the involvement of specific transport mechanisms. Some plant species including wheat, *Arabidopsis*, common bean (*Phaseolus vulgaris*) and soybean have been identified to exude more than one OA in response to Al exposure (**Table 1**). Although the mechanism of simultaneous release of OA in response to Al remains unknown, this indicates that multi-transport response and co-expression may function in these plants. Several studies have revealed that the time for Al-induced OA secretion varies from plant to plant (Kichigina et al., 2017; Silva et al., 2018; Ma et al., 2020b). Based on the duration and rate of OA released after Al exposure, two patterns of Al-induced secretion of OA have been proposed (**Figure 2B**). In pattern I, no obvious delay in time and rate of OA secretion was observed upon Al exposure (**Figure 2B**). Such a pattern occurs within minutes of Al exposure and has been observed in buckwheat, wheat, barley and beets (Zheng et al., 2005; Gruber et al., 2010; Li et al., 2017; Silva et al., 2018). The rapid OA secretion could suggest that Al interacts with a pre-existing anion channel and require no induction of transporter genes. However, in pattern II, there is a notable delay in time and rate of OA secretion after Al exposure (**Figure 2B**). OA secretion may delay by several hours to days and the rate of secretion may increase with the time of Al exposure. For example, In poplar, citrate secretion was induced 12−24 hours after Al treatment (Li et al., 2017) while malate secretion in *Hevea brasiliensis* occurred 2−5 days after Al treatment (Ma et al., 2020b). A similar Al-induced OA secretion pattern has been observed in maize (Du et al., 2021), rice (Yokosho et al., 2016a), sorghum (Sivaguru et al., 2013), rye (Silva et al., 2013) and *Arabidopsis* (Liu et al., 2009).

**Table 1:** Different plants secretes different organic acids.

Plant species	OA type Release	Transporter Gene(s)	Reference
*Amaranthus hypochondriacus*	Citrate and Oxalate	Unknown	(Fan et al., 2016)
*Arabidopsis thaliana*	Malate and Citrate	*AtALMT1* and *AtMATE1*	(Liu et al., 2009)
*Brassica napus*	Malate	*BnALMT1/2*	(Ligaba et al., 2006)
*Brassica Oleracea*	Malate	*BoALMT1*	(Zhang et al., 2018)
*Cajanus cajan*	Citrate	*CcMATE4*	(Dong et al., 2019)
*Callisthene fasciculata*	Citrate and Oxalate	Unknown	(de Souza et al., 2020)
*Camelia sinensis*	Oxalate	Unknown	(Devi et al., 2020)
*Camelina sativa*	Malate	*CsALMT1*	(Park et al., 2017)
*Citrus sinensis*	Citrate	Unknown	(Yang et al., 2012)
*Eucalyptus camaldulensis*	Citrate	*EcMATE1*	(Sawaki et al., 2013)
*Fagopyrum esculentum*	citrate	*FeMATE1; FeMATE2*	(Lei et al., 2017b)
*Fagopyrum esculentum*	Oxalate	Unknown	(Wang et al., 2015a)
*Glycine max*	Citrate and Malate	*GmMATE and GmALMT1*	(Liang et al., 2013; Liu et al., 2016b)
*Glycine soja*	Citrate	*GsMATE*	(Ma et al., 2018)
*Gossypium hirsutum*	Citrate	*GhMATE1*	(Kundu and Ganesan, 2020)
*Hevea brasiliensis*	Malate	*HbALMT1, HbALMT2, HbALMT13, and HbALMT1*	(Ma et al., 2020b)
*Hordeum vulgare*	Mate and Citrate	*HvAACT* and *HvALMT1*	(Furukawa et al., 2007; Gruber et al., 2010)
*Lupinus albus*	Malate	*LaALMT1*	(Zhou et al., 2020)
*Medicago sativa*	Malate	*MsALMT1*	(Chen et al., 2013)
*Oryza sativa*	Citrate	*OsFRDL2, OsFRDL4*	(Yokosho et al., 2016a; Li et al., 2018a; Huang et al., 2019)
*Phaseolus vulgaris*	Citrate	*PvALMT* and *PvMATE*	(Njobvu et al., 2020)
*Pisum sativum*	Citrate	Unknown	(Kichigina et al., 2017)
*Populus trichocarpa*	Citrate and Malate	*PoptrMATE54 and PoptrALMT10*	(Cardoso et al., 2020)
*Populus trichocarpa*	Citrate	*PtrMATE1; PtrMATE2*	(Li et al., 2017)
*Secale cereale*	Malate and Citrate	*ScALMT1; ScFRDL2*	(Collins et al., 2008; Liu et al., 2009; Santos et al., 2018)
*Secale cereale*	Oxalate	Unknown	(de Sousa et al., 2016)
*Solanum lycopersicum*	Malate	*SlALMT3, SlALMT9*	(Ye et al., 2017; Wang et al., 2020b)
*Sorghum bicolor*	Malate	*SbMATE1*	(Sivaguru et al., 2013; Melo et al., 2019)
*Triticum aestivum*	Malate and citrate	*TaALMT1 and TaMATE1, TaMATE1B*	(Garcia‐Oliveira et al., 2014; Silva et al., 2018; Liu et al., 2021)
*Vigna umbellata*	Citrate	*VuMATE1*	(Liu et al., 2016c)
*Zea mays*	Citrate	*ZmMATE1*	(Maron et al., 2013; Du et al., 2021)

Comparative studies of Al tolerant varieties were used to identify genes encoding malate and citrate transporter (Kochian et al., 2015). Previous studies have identified Al-activated malate transporter (ALMT) in wheat (*TaALMT1*) (Sasaki et al., 2004) which encodes an Al^3+^-activated anion channel on the plasma membrane for malate efflux and the multidrug and toxic compound extrusion (MATE) family of genes comprising of sorghum (*Sorghum bicolour*) (*SbMATE*) (Magalhaes et al., 2007) and barley (*Hordeum vulgare*) aluminium-activated citrate transporter 1 (*HvAACT1*) (Furukawa et al., 2007) that encode plasma membrane citrate transporters at the root apex. These transporters release specific OA from plant roots into the rhizosphere in response to Al exposure. Silva et al. (2018) indicated that the *ALMT1* gene is made up of five intron and six exons and is not related to the *MATE* gene (Upadhyay et al., 2019; Wang et al., 2020a), suggesting that Al-tolerance in sorghum and wheat evolved independently and that distinctly different genes encode the same physiological response to Al-tolerance. The homolog of these genes has recently been cloned in other crops (**Table 1**) which indicates that the primary determinant for the type of OA to be secreted depends on the plant species and the family of transporter genes expressed.

Moreover, accumulating evidence indicates that not all *ALMT* and *MATE* genes are involved in Al-tolerance but perform other physiological functions (Sharma et al., 2016; Sasaki et al., 2016; dos Santos et al., 2017). For instance, in Arabidopsis, four members of ALMT (*AtALMT4, AtALMT6, AtALMT9* and *AtALMT12*) (Liu and Zhou, 2018) mediate guard cell regulation while in tomato, two members (*SIALMT4* and *SIALMT5*) are involved in fruit development and seed OA content (Sasaki et al., 2016). Likewise, *VvMATE1* and *VvMATE2* are involved in proanthocyanin transport in *Vitis vinifera* fruit development (Pérez-Díaz et al., 2014), while in rice, *OsMATE2* regulate Arsenic uptake (Das et al., 2018). Similar physiological and developmental function of ALMT and MATE other than Al-tolerance has been reported in *Vitis vinifera* (De Angeli et al., 2013), *Hordeum vulgare* (Xu et al., 2015), strawberry (Chen et al., 2018b), blueberry (Chen et al., 2015) and *Lotus japonicus* (Takanashi et al., 2016). Nonetheless, transporters for oxalate efflux are still elusive even though it was shown to be involved in Al-tolerance (Wang et al., 2015a). Although the mechanism of how Al-induced anion channels remain unclear and is still a major debate among researchers, three possibilities have been proposed to describe the patterns of Al-induced OA secretion (Ma et al., 2001): (1) Al directly interacts with anions channels and opens it for OA efflux; (2) Al is sensed by a specific plasma membrane receptor (unknown) and through cytoplasmic transduction pathways activate the anion channels for OA efflux. (3) Al enters the cell by unknown means and interacts directly or indirectly with anions efflux channels. However, Wang et al. (2022) used cryo-electron microscopy and electrophysiological measurements to reveal that AtALMT1 is composed of six transmembrane helix (TM) and six cytosolic α-helices. Al binds to the external structure on AtALMT1 which triggers changes in the TM1-2 loop and TM5-6 loop conformational resulting in the opening of the anion gate for malate efflux (Wang et al., 2022). This suggests that Al indeed interacts with anion channels to secrete OA for external Al detoxification (Pattern I, **Figure 2B**). Nevertheless, it is relatively unknown whether this mechanism is the same for other plant species.

#### 3.1.2 Release of other organic compounds

Some plants do not only exude organic acids to chelate Al ions but other secondary metabolites including benzoxazinoids and phenolics compounds have been reported (Morita et al., 2011; Maejima et al., 2017; Zhao et al., 2019). In *C. sinensis*, Morita et al. (2011) indicated that caffeine, a phenolic compound, was released by the roots in response to Al exposure. In Al tolerant species, Maejima et al. (2017) showed that *Melastoma malabathricum* and *Melaleuca cajuputi* roots produced higher phenolic content in their roots which could chelate Al ions. Similarly, *Eucalyptus camaldulensis* produces oenothein B in its roots in response to Al exposure which detoxifies external Al and thereby, promoting Al tolerance (Tahara et al., 2014). Intriguingly, recent studies demonstrated that Al-tolerant maize roots secrets two benzoxazinoids such as DIMBOA (2,4-dihydroxy-7-methoxy-1,4-benzoxazin- 3-one) and MBOA (6-methoxy-benzoxazolin-2-one) to prevent Al entry into the root cells (Guimaraes et al., 2014; Zhao et al., 2019). *In vitro* study indicated that both DIMBOA and MBOA chelates Al and form a non-toxic DMBOA-Al and MBOA-Al complexes in the rhizosphere (Zhao et al., 2019). Although secondary compounds are known to be less effective chelators compared to OAs, it remains unknown how phenolics and benzoxazinoids are secreted in response to Al exposure. Additionally, regulatory and transporter genes involved in their release have not yet been identified and still unclear whether other plant species may utilize this mechanism.

#### 3.1.3 Secretion of root mucilage and formation of border cells

Exudation of mucilage and formation of border cells around the root apex is an important exclusion mechanism employed by some plant species to thrive in Al toxic environment (Cai et al., 2013; Okamoto and Yano, 2017; Feng et al., 2019). In several plant species, the root border cells (RBCs) serve as a protective shield between the root apex and Al toxicity by enhancing the packing of mucilage (Cai et al., 2013). Mucilage is a gel-like polysaccharide material that is secreted from the root cap and border cells (Cai et al., 2013). It is rich in negatively charged carboxyl compounds including uronic acids, that immobilize toxic metal cations and render them non-toxic to plants (Watanabe et al., 2008b; Watanabe et al., 2008a). Roots of *M*. *malabathricum* exude mucilage which facilitates Al accumulation and its removal reduced Al binding (Watanabe et al., 2008b; Watanabe et al., 2008a). Similarly, Okamoto and Yano (2017) found that maize seedlings secret mucilage in their root tips and its removal enhances rapid recovery which lowered Al accumulation. Interestingly, Yang et al. (2016a) showed that Al accumulates in the akali-soluble pectin of RBC due to an increase in uronic acid content which chelates Al and inhibits its entry into the root apiece of pea. A similar Al exclusion effect of RBC has been observed in rice (Cai et al., 2012; Nagayama et al., 2019), castor (*Ricinus communis*) (Alves Silva et al., 2014), and soybean (Cai et al., 2012). However, the removal of RBCs from root apex increased Al sensitivity and accumulation in rice (Cai et al., 2012), soybean (Cai et al., 2013) and pea (Yang et al., 2016a). These suggested that RBCs are a vital Al exclusion mechanism in these plant species. Recently, Feng et al. (2019) reported that the RBCs surface of pea roots possess a layer of silica nanoparticles, which serves as an external Al-resistant covering that chelate Al in the apoplastic space and restrict its buildup in the cytoplasm. Nevertheless, it remains unknown how this mucilage is secreted and whether its secretion and RBC formation are conserved in plants.

#### 3.1.4 Increase in rhizosphere pH

Rhizosphere pH is crucial for the solubility of Al and the toxic effect of Al on plants and it is viewed as an Al exclusion mechanism (Yang et al., 2011d). In response to Al toxicity, some plants have evolved strategies to increase their rhizosphere pH, which was shown to decrease Al solubility and activity (Bose et al., 2010; Yang et al., 2011d; Wang et al., 2015a). Thus, restricting Al entry into the root cell and promoting Al tolerance (Kochian et al., 2015). Al-tolerant wheat varieties and other plant species elevate rhizosphere pH (from 4.5 to 4.8 in wheat treated plants *via* the increased influx of H^+^ ions (Bose et al., 2010; Yang et al., 2011d; Wang et al., 2015a). Moreover, exudation of OA to the rhizosphere in response to Al toxicity can alter rhizosphere pH (Javed et al., 2013). Although this is not well investigated, secretion of OA has been reported to stimulate the influx of H^+^ ions that can increase rhizosphere pH (Yang et al., 2011b; Wang et al., 2015a). The involvement of PM H^+^-ATPase has been demonstrated to play a vital role in modulating rhizosphere pH (Bose et al., 2010; Yang et al., 2011d). In Al-tolerant wheat, PM H^+^-ATPase activity was considerably higher and correlated with an increase in rhizosphere pH compared to the Al-sensitive variety (Yang et al., 2011d). Furthermore, Li et al. (2018b) revealed that the root surface pH of a pea plant was increased by regulation of PM H^+^-ATPase activity and polar auxin transport which reduced Al-accumulation in the root.

### 3.2 Internal tolerance mechanism

#### 3.2.1 Cell wall modification

Root cell wall is the principal barrier against harmful environmental cues and is considered a major target of Al (Yang and Horst, 2015; Geng et al., 2017; Zhu et al., 2017; Wu et al., 2022). Root cell walls are rich in carboxylic material including pectin, cellulose and hemicellulose, which possess a high affinity for Al ions (Geng et al., 2017; Wu et al., 2022). Adsorption capacity study in *Arabidopsis* revealed that hemicellulose in root cell wall exhibits the highest affinity compared to pectin and was suggested as the core target of Al (Yang et al., 2011a). Several studies have shown that alteration in cell wall composition is crucial for enhancing Al tolerance (Wang et al., 2015c; Yan et al., 2018; Xu et al., 2018; Riaz et al., 2019; Xu et al., 2019; Zhu et al., 2019). Such modifications in cell wall structures are promoted by a complex network of cell wall enzymes including xyloglucan endotransglucosylase (XET), xyloglucan endotransglucosylase-hydrolase (XTHs), pectin methylesterases (PME) and expansin (Zhu et al., 2012; Yang et al., 2013; Kochian et al., 2015). *XTH* genes encode xyloglucan endohydrolase (XEH) and XET that catalyse the formation of cellulose-hemicellulose (xyloglucan) matrix and contribute to cell wall expansion (Zhu et al., 2012). In *Arabidopsis*, Al reduced the expression and activity of XTH17 and XTH31 in root cells whereas mutants of *XTH17* and *XTH31* exhibited improved Al tolerance by lowering hemicellulose content and retaining less Al in their cell wall (Zhu et al., 2014) Similarly, in *Phaseolus vulgaris*, expression of *XTH* genes and *XET* activities significantly reduced Al binding by changing cell wall porosity in root tips and enhancing Al tolerance (Zhang et al., 2016).

Cell wall pectin content and methylation influence the degree of Al tolerance (Yang et al., 2013). Yang et al. (2011c) reported in buckwheat (*Fagopyrum tataricum*) that higher Al accumulation in Al-sensitive cultivar is facilitated by pectin content rather than the degree of methylation. The Al-sensitive cultivar exhibited a greater sensitivity of pectin methylesterases activity to Al which resulted in a significant increase in low-methyl-ester pectins and decrease of high-methyl-ester pectins (Yang et al., 2011c). Li et al. (2016b) observed that pectin content increased in Al-sensitive than Al-tolerant pea cultivar and Al PME activity was enhanced in the sensitive cultivar which resulted in higher demethylesterified pectin content thereby enhancing Al accumulation in the root cell wall. Moreover, higher demethylated pectin mediated by PME results in the formation of negatively charged demethylesterified pectin and leads to an increase in Al ion binding (Li et al., 2016b). These suggest that Al-tolerant cultivars exhibit low PME activity and increase methylated pectin content. Furthermore, pectin methylesterase genes have been revealed to contribute to Al tolerance in several plant species. In *Arabidopsis*, *PME46* was reported to enhance Al tolerance by reducing PME activities and decreasing Al binding to cell walls (Geng et al., 2017). Unlike other PMEs, *PME46* has an PME inhibitor domain (N-terminal pro region) which promotes unprocessed PMEs retention and represses PME enzyme activity suggesting that PME46 activity could activate transcriptional repression of other *PMEs* thereby facilitating the accumulation of methylated pectin in the cell wall (Geng et al., 2017). Methylated pectin exhibits lower negative charge which will bind less Al and promote Al tolerance. Also, in alfalfa (*Medicago sativa*), polygalacturonase genes, *MsPG1* and *MsPG4*, were reported to increase Al tolerance by enhancing cell wall plasticity and porosity and reducing Al accumulation in the cell wall (Li et al., 2020; Fan et al., 2022).

Molecular evidence revealed that in rice, *OsSTAR1* (Sensitive to Aluminum Rhizotoxicity 1) and *OsSTAR2* (Sensitive to Aluminum Rhizotoxicity 2) genes encode a bacterial-type ATP binding cassette (ABC) transporter protein for cell wall modification during Al toxicity. OsSTAR1 and OsSTAR2 interact to form a vesicle membrane-localized complex in root cells that export UDP-glucose from the cytoplasm into the cell wall perhaps through vesicular exocytosis (Huang et al., 2009). These compounds could bind to Al in the apoplast or utilize as substrates by cell wall modifying enzymes and thereby, reducing Al binding and damage to the cell wall (Buchanan et al., 2015). Recently, STAR1 and STAR2 were functionally characterised in buckwheat to regulate Al tolerance (Che et al., 2018b; Xu et al., 2018; Xu et al., 2019) and SbSTAR1 in sorghum (Gao et al., 2021). Similar to OsSTAR1/OsSTAR2 complex, FeSTAR1 and FeSTAR2 form an ABC transport protein that export UDP-glucose which influence hemicellulose metabolism by modulating XET activities (Xu et al., 2018; Xu et al., 2019). Additionally, SbSTAR1 enhanced Al tolerance when expressed in *Arabidopsis* lines (Gao et al., 2021). Although the counterpart of SbSTAR1 had not been characterised, it was suggested that it could mediate Al resistance *via* modulation of hemicellulose content in the root cell wall. These suggest that STAR1 and STAR2-mediated Al tolerance could be a conserved mechanism in plants although not yet identified in other plant species. Nevertheless, it remains unclear how the UDP-glucose exactly mediates Al tolerance and how it influences XET activity.

#### 3.2.2 Internal detoxification and compartmentalization

Studies on Al tolerance mechanisms in Al-resistant plants and/or Al-hyperaccumulators have increased our understanding of how these plants detoxify Al within their roots (Kochian et al., 2015). Al-resistant plant species including hydrangea, *melastoma malabatbricum*, buckwheat and black tea (*Camellia sinensis*) can take up, detoxify and accumulate relatively high content of Al in their leaf tissues without displaying Al toxicity effects (Wang et al., 2015b; Bojórquez-Quintal et al., 2017). Evidence revealed that internal detoxification of Al is mediated by intracellular Al chelation and compartmentalization of Al-OA complexes into vacuoles (**Figure 2C**). In buckwheat, Al is taken up into the root cell and forms a non-toxic complex with oxalate at a 1:3 (Al-oxalate) ratio (Klug and Horst, 2010). During Al transport from root to shoot, the Al-oxalate is loaded into the xylem sap where Al-oxalate is exchanged for Al-citrate (1:1). In the leaves, Al-citrate is converted to Al-oxalate and sequestered into the vacuoles (Wang et al., 2015b). Although the mechanism of ligand exchange during xylem loading and unloading is unknown, a similar Al-detoxification mechanism was observed in *melastoma malabatbricum* and *C. fasciculata* (de Souza et al., 2020). Moreover, Al forms a no-toxic complex with catechin in the leaves of tea plants (Fu et al., 2020), whereas, in hydrangea, Al is complexed with 3-caffeoylquinic and delphinidin 3-glucoside in the sepals and citrate in the leaves (Ma et al., 1997). In *Andropogon virginicus*, a wild species of Poaceae, Al is accumulated in the leaf’s trichomes and spikes of which some Al portions are secreted as viscous sap from the trichome apex (Ezaki et al., 2013).

##### 3.2.2.1 Transporters for internal Al tolerance

Several studies have identified and functionally characterized transporter genes that mediate internal Al detoxification and sequestration. In rice, natural resistance-associated macrophage protein (Nramp) Al transporter 1 (OsNrat1) encodes a unique plasma membrane transporter that mediates the influx of Al ions and contributes to Al tolerance in rice (Xia et al., 2010; Li et al., 2014). This high influx of Al ions mediated by OsNrat1 could reduce Al levels in the root cell wall by transporting Al into the root cells and subsequently sequestered into the vacuole thus promoting Al tolerance (Huang et al., 2012). Similarly, ALS1 (Aluminum sensitive 1) is an ABC transporter that is localized on the tonoplast and crucial for vacuolar Al sequestration and internal detoxification of Al in rice (Huang et al., 2012), *Arabidopsi*s (Larsen et al., 2007; Kochian et al., 2015), and buckwheat (Lei et al., 2017a). *AtALS1* is constitutively expressed in the roots and vasculature of Arabidopsis (Larsen et al., 2007) while in rice while *OsALS1* is expressed in the roots and induced by Al exposure (Huang et al., 2012). However, in buckwheat, *FeALS1.1* expression is upregulated by Al in both roots and leaves whereas *FeALS1.2* is not affected by Al (Lei et al., 2017a). Although *Nrat1* has not yet been characterized in other plant species, the similarities in localization and expression pattern of *OsALS1* and *OsNrat1* (Xia et al., 2010; Huang et al., 2012) could suggest that these transporters act collectively to mediate the internal detoxification of Al in rice. In buckwheat, FeIREG1 which belongs to the IRON REGULATED/ferroportin (IREG) transporters, is located in the tonoplast and sequesters Al into root vacuoles to enhance internal Al tolerance (Yokosho et al., 2016b). Similarly, FeIREG1 homolog has been characterized in soybean (GmIREG3) and overexpression of *FeIREG1* and *GmIREG3* in Arabidopsis promotes Al tolerance (Yokosho et al., 2016b; Cai et al., 2020). Moreover, Negishi et al. (2012) demonstrated in hydrangea (*Hydrangea macrophylla*) roots that two members of the aquaporin family, plasma membrane Al transporter 1 (PALT1) and vacuolar Al transporter (VALT), mediate cytosolic Al influx and subsequent sequestration into the vacuoles, respectively. Nevertheless, it remains unknown which Al form is transported by these two aquaporins. Similarly, Wang et al. (2017) revealed that a plasma membrane-localized member of the nodulin 26-like intrinsic protein (NIP) plays a critical role in Al uptake and internal tolerance mechanism in Arabidopsis. NIP1;2 is an Al-malate transporter which mediates the removal of Al from root cell walls into the cytosol and facilitates xylem loading and root-to-shoot translocation of Al-malate (Wang et al., 2017). Additionally, the function of NIP1;2 depends on an operational Al-induced malate transporter, which is mediated by AtALMT1 in *Arabidopsis* roots. Besides, NIP1;2 and ALMT1 exhibit an epistatic association, which suggests a coordinated expression and that both NIP1;2 and ALMT1 act in the same pathway to mediate Al tolerance in *Arabidopsis* (Wang et al., 2020a). Therefore, effective coordination between Al exclusion and the internal tolerance mechanism is paramount to attaining Al tolerance in *Arabidopsis*.

##### 3.2.2.2 Transcriptional regulation of Al tolerance in Plants

**Figure 3:**
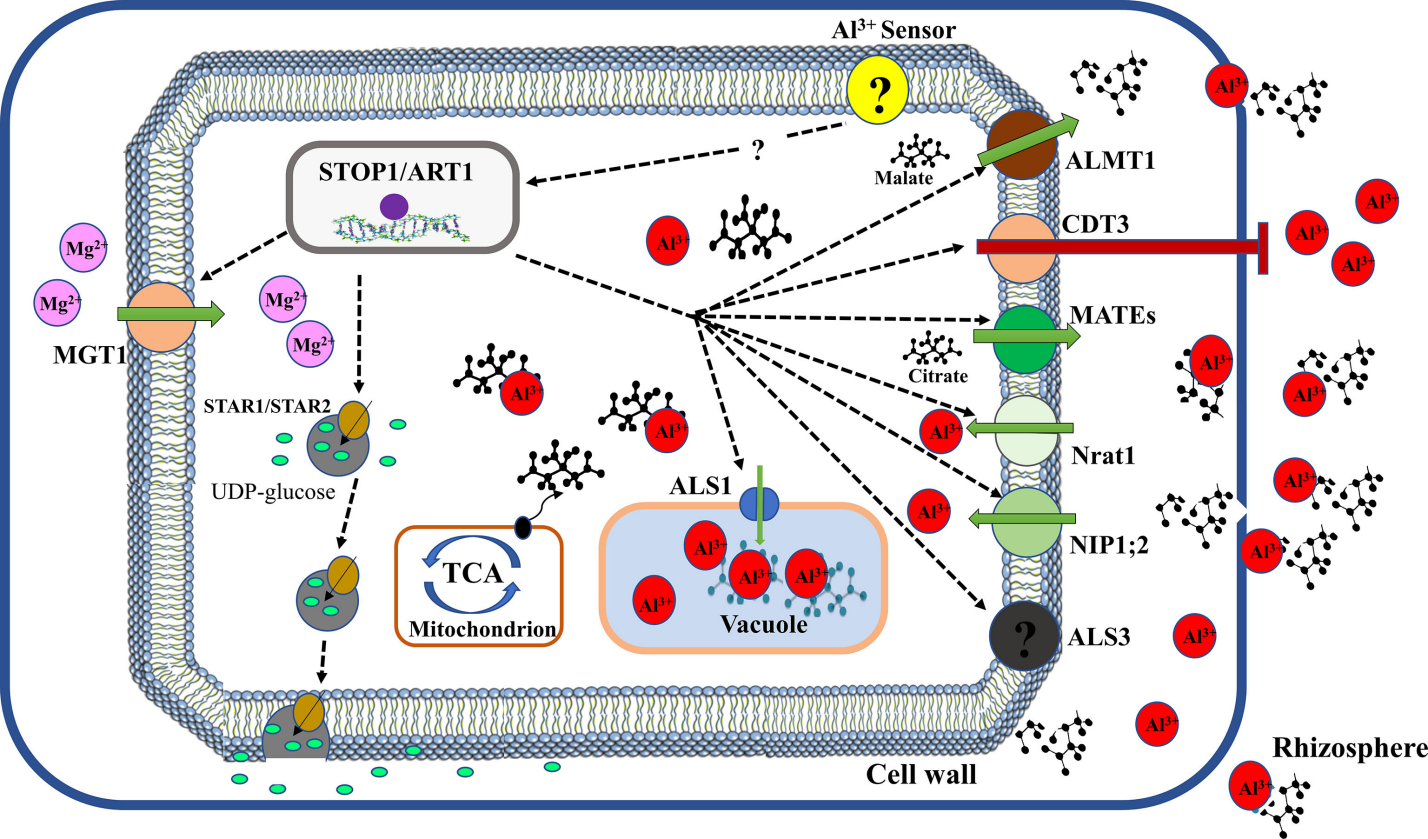
Transcriptional regulation of Al tolerance in plants. Al-stress is sensed by an unknown sensor and triggers activation of a C2H2-type zinc finger transcription factor (STOP1 or ART1) to initiate downstream signalling of Al tolerance genes. ALMT1, aluminum-activated malate transporter 1 for malate transport; CDT3, cadmium transport 3 block Al entry; MATEs, multidrug and toxic compound extrusion for citrate transport (e.g. OsFRDL4 and OsFRDL2 in rice, AtMATE1 in Arabidopsis); Nrat1, natural resistance-associated macrophage protein (Nramp) Al transporter 1 facilitate Al influx; NIP1;2, plasma membrane-localized nodulin 26-like intrinsic protein transport Al-malate transporter in their cell; ALS3, Aluminum sensitive 3 promote Al influx; ALS1, aluminum sensitive 1 enhance sequestration of Al-OA complex into the vacuole; TCA, tricarboxylic acid cycle mediate OA production; STAR1/STAR2, sensitive to aluminum rhizotoxicity 1/2 facilitate UDP-glucose transport; MGT1, magnesium transporter 1 for magnesium influx.

Mutational and molecular analyses have provided compelling evidence that Al activates coordinate expression of Al-tolerant genes (**Figure 3**). Several transcription factors have been reported to regulate the expression of downstream genes required to enhance Al tolerance. In *Arabidopsis*, a C2H2 zinc finger transcription factor, sensitive to protein rhizotoxicity 1 (STOP1) localised in the nucleus was reported to play a critical role in Al tolerance (Iuchi et al., 2007). AtSTOP1 modulate the expression of *AtALMT1via* direct binding to consensus sequences in its promoter region (Tokizawa et al., 2015). Additionally, AtSTOP1 was reported to control the expression of *AtMATE* and *ALS3* to mediate Al tolerance (Kochian et al., 2015). Nevertheless, it remains unknown whether AtSTOP1 directly interact with these genes. Moreover, Al exposure was showed not to affect the expression of AtSTOP1 but stimulate the expression of several AtSTOP1 regulated downstream genes (Iuchi et al., 2007; Sawaki et al., 2009) suggesting that Al post-transcriptionally modulates AtSTOP1. Intriguingly, it was revealed that Al stress stimulates the build-up of AtSTOP1 proteins and F-box proteins, REGULATION OF ATALMT1 EXPRESSION 1 (RAE1) and RAE1 homolog 1 (RAH1) which can interact with STOP1 proteins to mediate its degradation *via* the ubiquitin– 26S proteasome pathway (Zhang et al., 2019c; Fang et al., 2021b). Similarly, Guo et al. (2020) reported that HPR1, which encodes a constituent of the THO/TREX complex reduces AtSTOP1 protein accumulation by regulating nucleocytoplasmic export of AtSTOP1 mRNA. Furthermore, Fang et al. (2021a) demonstrated that SUMO E3 ligase SIZI partially modifies AtSTOP1 proteins *via* SUMOylation to modulate AtSTOP1 functions. These studies suggest that both post-transcriptional and post-translational mechanisms could regulate AtSTOP1 stability and function.

In rice, a homolog of AtSTOP1, OsART1 (Al Resistance Transcription factor 1) is localized in the nucleus and performs a comparable role in Al tolerance (Yamaji et al., 2009). Like AtSTOP1, OsART1 regulate numerous Al-responsive genes involved in exclusion and internal Al tolerance mechanisms. These genes include *OsFRDL2*, *OsFRDL4*, *OsNrat1*, *OsSTAR1*/*OsSTAR2* complex, *OsALS1*, *OsART2*, *OsMGT1*, *OsEXPA10* and *OsCDT3* (Huang et al., 2009; Chen et al., 2012; Xia et al., 2013; Yokosho et al., 2016a; Che et al., 2016; Lu et al., 2018; Che et al., 2018a). Moreover, OsCDT3 encodes a predicted cysteine-rich peptide protein located at the plasma membrane of rice root cells which directly binds Al and restricts Al entry into root cell, thus contributing to Al tolerance in rice (Xia et al., 2013). *OsEXPA10* is an expansin gene whose expression is induced by Al and required for root cell expansion during Al stress (Che et al., 2016). However, its role in rice high Al tolerance is minimal. Recently, a homolog of OsART1, OsART2 was shown to be up-regulated by Al and regulate Al tolerance in rice as *osart2* knockout lines exhibit hypersensitivity to Al (Che et al., 2018a). Transcriptomic analysis indicated that OsART2 regulation of genes does not overlap with ART1 genes but modulates four genes which are implicated in Al tolerance. This suggests that OsART1 and OsART2 regulate different Al tolerance pathways and the latter could play an additional role in rice Al tolerance. Unlike AtSTOP1, the regulation mechanism of OsART1 stability and function remains unknown.

Furthermore, AtSTOP1/OsART1 homologs have been identified in several plant species including pigeon pea (CcSTOP1) (Daspute et al., 2018), sorghum (SbSTOP1) (Gao et al., 2019), tobacco (NtSTOP1) (Ito et al., 2019), cotton (GhSTOP1) (Kundu et al., 2019), rye (ScSTOP1) (Silva-Navas et al., 2021), soybean (GmSTOP1) (Wu et al., 2018; Zhou et al., 2018), tea (CsSTOP1) (Zhao et al., 2018), rice bean (VuSTOP1) (Fan et al., 2015), tomato (SlSTOP1) (Zhang et al., 2022), barley (HvATF1) (Wu et al., 2020) and wheat (TaSTOP1) (Garcia-Oliveira et al., 2013). Nevertheless, it remains unknown how Al stress transduces the signal to activate and stabilize STOP1/ART1.

#### 3.2.3 Osmolytes accumulation

Production and build of osmolytes or compatible solutes are biochemical mechanisms employed by several plants under Al stress to promote retention of water status, transfer of cellular energy, macromolecules and membrane stabilization and ROS scavenging and thereby, contributing to the maintenance of cellular homeostasis (Pandey et al., 2016; Ejaz et al., 2020). Osmolytes are water-soluble uncharged molecules, which include amino acids (e.g. proline, γ-aminobutyric acid), sugars (trehalose, sucrose, glucose) and ammonium compounds (glycine betaine, choline, proline betaine) and whose accumulation do not interfere with cellular functions (Ejaz et al., 2020). In buckwheat, proline and total sugar content increased proportionally to Al concentration and was implicated in Al tolerance (Pirzadah et al., 2019). In two rye plants with different Al tolerance, de Sousa et al. (2020) demonstrated that proline content increased by threefold and 20% in Al-tolerant and Al-sensitive lines respectively due to the regulation of proline biosynthetic pathways which include enhanced glutamate and ornithine pathways for proline biosynthesis and re-oxidation to 1-pyrroline-5-carboxylate. Moreover, Bera et al. (2019) revealed that rice plants accumulated high levels of glycine betaine in response to Al exposure. However, the activity of betaine aldehyde dehydrogenase, an important enzyme that mediates glycine betaine biosynthesis was considerably reduced which suggests that an unknown alternative pathway for glycine betaine production may function in rice to alleviate Al-induce osmotic stress. Similarly, the involvement of elevated osmolyte levels in response to Al exposure has been reported in alfalfa (Ma et al., 2020a), lettuce (Silva and Matos, 2016), rice (Awasthi et al., 2019) and sugarcane (Mantovanini et al., 2019). Furthermore, as Al toxicity induce water stress, these osmolyte function as osmoprotectants by reducing intracellular water potential, regulating turgor dynamics and stabilizing proteins and membrane integrity, thereby promoting Al tolerance (Ejaz et al., 2020).

#### 3.2.4 Production and activation of antioxidants

The alleviation of Al-induced excessive ROS accumulation *via* antioxidant production and associated enzyme activities in plant cells is one of the most characterized defense mechanisms reported in several plant species (Gill and Tuteja, 2010; Awasthi et al., 2019). This antioxidant defense pathway is categorized into the enzyme-mediated and non-enzymatic antioxidant systems. The enzyme-mediated defense system involves an increase in activities and accumulations of antioxidant enzymes including ascorbate peroxidase (APX), monodehydroascorbate reductase (MDHAR), dehydroascorbate reductase (DHAR), guaiacol peroxidase (GPX), peroxidases (POD), superoxide dismutase (SOD), catalase (CAT), and glutathione reductase (GR) and/or upregulation of antioxidant enzyme genes expression which is induced by Al stress in several plants and levels correlate to Al tolerance (Sharma et al., 2012; Yusuf et al., 2016; Liu et al., 2018; Awasthi et al., 2019; Du et al., 2020; Devi et al., 2020; Salazar-Chavarria et al., 2020). SOD is the first line of defence against oxidative stress, which catalyses the dismutation of O_2_^·-^ radicles into oxygen and H_2_O_2_. The H_2_O_2_ is subsequently reduced into water by APX, GPX, CAT and POD (Sharma et al., 2012). Reduction of H_2_O_2_ by APX is mediated by using ascorbic acid as an electron donor which is the first step of the ascorbate-glutathione cycle (Sharma et al., 2012). However, the non-enzymatic antioxidants which include ascorbate, carotenoid, phenolics, flavonoids and glutathione act together with the enzymatic antioxidants to detoxify ROS and promote Al tolerance (de Sousa et al., 2016; Maejima et al., 2017; Du et al., 2020; Ejaz et al., 2020). In two rye genotypes, ROS scavenging was mediated by GPX and POD in Al-sensitive lines while CAT catalyzed this function in Al-tolerant lines suggesting the role of different enzymes in ROS mitigation. In buckwheat cultivars, antioxidant activities were enhanced in an Al dose-dependent manner which correlated to significant Al tolerance (Pirzadah et al., 2019; Salazar-Chavarria et al., 2020). Similarly, antioxidant enzyme activities and antioxidant metabolites (glutathione disulfide, ascorbic acid, dehydroascorbate and reduced glutathione) contents were significantly increased in wheat roots exposed to Al stress (Yusuf et al., 2016; Liu et al., 2018). A similar conclusion was reported in rice (Awasthi et al., 2019; Ribeiro et al., 2022), watermelon (Malangisha et al., 2020), citrus (Yan et al., 2019), sorghum (Zhou et al., 2017), soybean (Zeng et al., 2020), maize (Du et al., 2021) and tomato (Ofoe et al., 2022) where Al stimulated the activities of antioxidant enzymes and promoted Al tolerance in these plants. These indicate that both enzymatic and non-enzymatic antioxidants detoxify Al-induce excessive ROS production and promote Al tolerance in plants.

#### 3.2.5 Hormonal regulation of Al stress

Phytohormones have been reported as key regulators of Al-induced root growth inhibition (Guo et al., 2014; Yang et al., 2017b; Yang et al., 2017a). In *Arabidopsis* roots, Yang et al. (2014) demonstrated that Al stress increases localized auxin biosynthesis in the root apex transition zone *via* the Trp aminotransferase 1 (TAA1)-dependent pathway. *TAA1* was upregulated in the root apex and mediated inhibition of root growth in response to Al treatment. Similarly, Liu et al. (2016a) recently reported that YUCCA (YUC), a flavin monooxygenase-like protein also modulates Al-induced localized auxin biosynthesis in *Arabidopsis* root apex transition and contributes considerably to root-growth inhibition under Al stress. This suggests that there could be other components of the Al-induced root growth inhibition pathway, which are yet to be identified. Moreover, an Al-induced increase in auxin accumulation in the root apex is regulated by auxin response factors (ARFs) (Yang et al., 2014; Liu et al., 2016a). In response to Al stress, ARFs control Al-induced root growth inhibition by modulating the expression of auxin signalling genes, IPT-dependent cytokinin biosynthetic genes and cell wall modification associated genes (Yang et al., 2014; Bai et al., 2017; Li et al., 2021). Besides, auxin act synergistically with ethylene to stimulate inhibition of root growth under Al stress (Yang et al., 2014; Liu et al., 2016a). Al-induced increased expression of 1-aminocyclopropane-1-carboxylic acid (ACC) oxidase (ACO) and ACC synthase (ACS) genes and enhanced ethylene biosynthesis (Yu et al., 2016). Tian et al. (2014) demonstrated that ethylene negatively regulates Al-induced malate exudation by aiming at TaALMT1 activities through an unknown mechanism. Heterologous expression of soybean ethylene response factor (*GsERF1*) in *Arabidopsis* enhanced ethylene and ABA-mediated Al tolerance by upregulating ACS genes and ABA-response genes (Li et al., 2022). Similarly, Chen et al. (2018a) revealed that the expression of soybean glycine-rich protein-like gene (*GmGRPL*) can promote Al tolerance by controlling auxin and ethylene levels in *Arabidopsis* roots. Interestingly, exogenous application of 6-benzylaminopurine and the use of cytokinin mutant lines showed that cytokinin work in synergy with auxin and act downstream of ethylene to promote Al-induced inhibition of root growth (Yang et al., 2017b). However, the understanding of cytokinin-mediated Al stress tolerance is limited.

ABA has been shown to regulate Al tolerance in plants. In buckwheat, Reyna-Llorens et al. (2015) reported that Al stress-induced endogenous accumulation of ABA which triggered the expression of *FeALS3*, contributing to Al tolerance. Similarly, ABA enhanced APX and CAT antioxidant activities in buckwheat seedlings to alleviate Al stress (Salazar-Chavarria et al., 2020). Promoter region analysis of VuMATE1 transport in rice revealed the presence of an ABA-responsive element which suggest that ABA could trigger citrate secretion under Al stress (Liu et al., 2016c). However, Fan et al. (2019b) recently indicated that Al stress triggers the endogenous accumulation of ABA in rice beans and that ABA-mediated Al stress tolerance is regulated by ABI5 which enhances cell wall modification and osmoregulation but not citrate efflux. In rice, an ABA stress and ripening genes (ASR) were reported to heighten Al stress tolerance by modulating the expression of *OsSTAR1*, *OsNrat1* and *OsFRDL4* (Arenhart et al., 2016). Also, Gavassi et al. (2021) found that Al stress induces 9-cis- epoxy carotenoid dioxygenase (NCED) gene expression which enhanced ABA biosynthesis in *Citrus limonia* roots and controlled leaf stomatal conductance.

Recently, Yang et al. (2017a) established that exogenous application of jasmonic acid (JA) promotes Al-induced root growth inhibition and that expression of CORONATINE INSENSITIVE1 (COI1) and MYC2, a JA receptor and a JA signalling modulator were up-regulated in response to Al stress. Additionally, melatonin has been reported to play a vital role in Al tolerance. In soybean, Zhang et al. (2017b) showed that melatonin content in roots increased with Al treatment which enhanced citrate and malate secretion as well as increased antioxidant activities. Similarly, exogenous application of melatonin enhanced Al tolerance in *Brassica napus* by increasing photosynthetic capacities and antioxidant activities (Sami et al., 2020). Moreover, Zhang et al. (2019b) showed that melatonin ameliorates Al-induced root growth reduction by interrupting nitric reductase- and nitric oxide synthase-dependent nitric oxide production which contributes to cell cycle progression and quiescent centre cellular activity. Furthermore, Sun et al. (2020a) established that exogenous application of melatonin considerably decreased cell wall polysaccharide content and pectin methylesterase activity and promote antioxidant enzyme activities to facilitate ROS scavenging and Al exclusion from root tips of wheat seedlings. However, it remains unclear how these phytohormones crosstalk with each other and other understudied plant hormones to induce root growth inhibition under Al stress. These studies indicate that plants respond to Al stress by regulating the biosynthesis, accumulation and distribution of various phytohormones which are involved in Al tolerance.

## 4 Conclusion and way forward

Aluminum is the third most widespread metal in the earth’s crust, and its impact on plants depends largely on concentration, exposure time, plant species, developmental age, and growing conditions (Huang et al., 1992; Bojórquez-Quintal et al., 2017; Aguilera et al., 2019; Ofoe et al., 2022). Beneficial effects of Al including stimulation of plant growth and mitigation of both biotic and abiotic stress have been reported in some plant species, especially in Al-tolerant species and when applied in lower concentrations (Andrivon, 1995; Arasimowicz-Jelonek et al., 2014; Wang et al., 2015b; Sun et al., 2020b; Zahra et al., 2021). However, it remains unknown how Al mediates this effect since its biological significance in cellular systems is still unidentified. Moreover, Al is generally considered a major limiting factor restricting plant growth and productivity in acidic soils. It instigates a series of phytotoxic symptoms in several Al-sensitive crops with inhibition of root growth and restriction of water and nutrient uptake as the obvious symptoms. In response to Al toxicity, most plants have evolved adaptive mechanisms including exclusion and internal tolerance to ameliorate Al phytotoxic effects. Although these mechanisms vary among plant species, they share close regulatory strategies. Therefore, studies on species-specific Al tolerance mechanisms will help identify new tolerant pathways in plants. Additionally, much progress has been made in recent years to understand the signalling and regulatory mechanisms of Al tolerance in plants. However, how plants sense Al toxicity and trigger downstream signalling cascades remain unknown and future studies focusing on the identification of plasma-membrane localised Al-receptor(s) and early signalling elements using molecular and reverse genetic approaches will help broaden our understanding of Al tolerance in plants. Furthermore, advances in sequencing several plant genomes and genome manipulation techniques now hold excellent promises for expediting the discovery of novel Al-tolerant genes and elucidation of novel mechanisms. The identification of Al-tolerant genes will enhance the development of Al-tolerant crops using molecular breeding and biotechnological techniques.

In the agricultural outlook, several strategies have been used to alleviate Al toxicity and enhance plant tolerance. Such strategies include liming, mineral nutrition, use of biostimulants and genetic engineering of Al-tolerant genes. Liming and mineral method reduces Al toxicity by increasing soil pH, but these are not economically feasible for small-scale farmers. Moreover, Al tolerance in several plants is regulated by multiple genes which mediate diverse signalling pathways that make it difficult to improve Al tolerance by transgenic approaches. Therefore, the development of multi-gene Al tolerant plants is crucial for enhancing crop productivity although there has been great opposition to transgenic crops in recent times (Nawaz et al., 2022). On the other hand, the use of biostimulants could be a sustainable strategy for improving plant growth and yield in acidic soils.

## Author contributions

RO: Conceptualization, Design, Writing - original draft, review and editing. SA: Writing – review & editing. GW-P: Writing – review and editing. BF: Validation, Writing – review and editing. RT: Supervision. LA: Conceptualization, Supervision, Validation, Writing – review and editing. All authors read and approved the final manuscript.
